# Mental Illness and Work-Related Limitations in Healthcare Workers: A Preliminary Retrospective Study

**DOI:** 10.3390/ijerph19159098

**Published:** 2022-07-26

**Authors:** Sara Gostoli, Laura Nicolucci, Carlotta Malaguti, Chiara Patierno, Danilo Carrozzino, Cristian Balducci, Sara Zaniboni, Vittorio Lodi, Carmine Petio, Chiara Rafanelli

**Affiliations:** 1 Department of Psychology, University of Bologna, 40127 Bologna, Italy; sara.gostoli2@unibo.it (S.G.); laura.nicolucci@studio.unibo.it (L.N.); chiara.patierno@studio.unibo.it (C.P.); danilo.carrozzino@unibo.it (D.C.); cristian.balducci3@unibo.it (C.B.); sara.zaniboni4@unibo.it (S.Z.); chiara.rafanelli@unibo.it (C.R.); 2 Department of Psychiatry, Bologna University Hospital Authority St. Orsola-Malpighi Polyclinic IRCCS, 40138 Bologna, Italy; carlotta.malaguti@studio.unibo.it; 3 Department of Management, Technology and Economics, ETH Zürich, 8092 Zürich, Switzerland; 4 Occupational Health Unit, Bologna University Hospital Authority St. Orsola-Malpighi Polyclinic IRCCS, 40138 Bologna, Italy; vittorio.lodi@aosp.bo.it

**Keywords:** clinimetrics, fit note, job limitation, occupational medicine, psychiatric diagnosis

## Abstract

This retrospective observational study investigated hospital staff requests for job fitness visits, addressed to occupational medicine. Specific objectives were to analyze: (1) health workers’ requests, sociodemographic characteristics, psychiatric diagnoses, assigned doctor’s fit notes, and (orthopedic, psychiatric) limitations; (2) associations between psychiatric diagnoses, sociodemographic (sex, age), and work-related (job, department) characteristics; (3) associations between the same psychiatric diagnoses/orthopedic limitations, fit notes, and/or psychiatric limitations. Data of St. Orsola-Malpighi Polyclinic health workers (N = 149; F = 73.8%; mean age = 48 ± 9.6 years), visited by both the occupational medicine physician and psychiatrist (January 2016–May 2019), were analyzed. 83.2% of the sample presented with at least one psychiatric diagnosis, including mood (47%), anxiety (13.4%), and anxious-depressive (10.7%) disorders. Significant differences between psychiatric diagnoses according to sex and fit notes (both *p* < 0.01) have been found, whereas no significant associations based on age and work-related characteristics have been observed. Analysis of frequencies of participants with the same psychiatric diagnosis (orthopedic limitation being equal), according to doctor’s fit notes and psychiatric work limitations, showed a high heterogeneity of assignments. The current occupational medicine procedure for fit notes/job limitations assignments does not allow taking into consideration clinical factors possibly associated with more specific assignments. To standardize the procedure and translate the psychiatrist’s clinical judgment into practice, further studies to test the usefulness of clinimetrics, which might represent a reliable approach in considering different fit notes and job limitations, are needed.

## 1. Introduction

Mental illness represents the second leading cause, after musculoskeletal disorders, of work-related problems, and it accounts for more than one-third of work-related illnesses [1]. Whatever the mental disorder, psychopathological conditions in work settings have deleterious effects on performance, absenteeism, and days of disability at work [2,3,4,5,6,7,8].

Hospital medical and technical staff may be exposed daily to health risks such as ergonomic hazards, shift work, work-related stress, and violence [9,10], which have been shown to cause mental disorders [11]. Among healthcare workers, both nurses [12,13,14,15] and physicians [16,17,18] may present high levels of stress and work-related mental illness [19,20]. Moreover, working in different departments, such as oncological and hematological, anesthesia and emergency areas, and intensive and critical care units, was associated with different levels of work-related stress and the presence of mental disorders [21,22,23,24,25,26,27].

In recent years, the mental health of healthcare workers has been gaining increasing attention worldwide as a major public health threatening issue, in terms of quality of care delivery [28]. There is strong evidence that healthcare workers are exposed to multiple stress factors within their work, which have the potential to negatively affect their physical and psychological well-being [29,30,31]. Even before the pandemic, indeed, in the United States, 44% of physicians reported at least one symptom of burnout in 2017 compared with about 54% in 2014 and about 45% in 2011 [32], whereas in Latin American countries, the mental health of healthcare workers was poorer than the mental health of the general population [33]. In Great Britain, the category of human health and social work activities had significantly higher rates of work-related stress, depression, or anxiety (2770 cases per 100,000 workers) than the average for all other industries [34]. According to the National Institute of Statistics, in Italy, 32% of healthcare workers reported exposure to at least one work-related psychosocial risk factor [35]. The World Health Organization estimates a projected shortfall of 18 million health workers by 2030, mostly in low- and lower-middle-income countries [36]. Given this international overview, the role of occupational medicine, its assessment of disability and psychological limitations at work, and, consequently, of psychic fitness for work, become crucial [37,38]. In Italy, the occupational physician’s assessment of fitness for work can be described by a categorization including standardized definitions, as follows: fit, fit with restrictions (in this case, the physician should indicate the type of limitation and whether it is temporary or permanent), temporarily unfit (in this case, the physician should specify a date for the follow-up), and permanently unfit [39]. The occupational physician evaluates whether cognitive, relational, behavioral, and creative skills have been compromised [40]. Based on this, he or she will formulate a fitness for work assessment explaining the state of health of the subject, considering the ability to carry out the work without worsening the worker’s own mental health and causing damage to third persons. Furthermore, the physician should be equipped with reliable instruments to evaluate cognitive and emotional abilities essential for the job [37]. For the collection of anamnestic data, it would be desirable to use structured and semi-structured interviews, which should also evaluate individual vulnerability with a certain sensitivity towards psychopathology [41]. The occupational physician should collaborate with other healthcare professionals (such as psychiatrists and psychologists) to manage possible mental problems [40] and he/she should assess whether the use of psychodiagnostic tests is necessary to formulate a complete worker’s clinical picture [42]. However, the overall Italian situation is far from optimal [40]. Moreover, in occupational medicine, little research was published on the assessment of fitness for work [43]. Porrone and Esposito [39] also pointed out, with reference to the Italian literature, that specific studies on fitness for work, including validated procedures and instruments for a standardized assessment of possible work-related pathological conditions, are insufficient. In the same vein, Serra and colleagues [44] showed that the assessment instruments in work contexts should be specific whereas there is very little scientific evidence, probably because standard or valid methodologies for all professions and circumstances are lacking, despite occupational specialists expressing this need.

The aims of the present preliminary retrospective observational study are as follows: (1) to assess healthcare workers’ requests, sociodemographic characteristics, psychiatric diagnoses according to current nosography, assigned doctor’s fit notes, and limitations (orthopedic and psychiatric) in an Italian hospital; (2) to identify statistical associations between psychiatric diagnoses, sociodemographic characteristics (e.g., sex, age), and work-related variables (e.g., job, department); (3) to evaluate descriptive associations between the same psychiatric diagnoses/orthopedic limitations, fit notes, and psychiatric limitations.

## 2. Materials and Methods

### 2.1. Sample

The sample included 149 healthcare workers at Sant’Orsola-Malpighi Polyclinic in Bologna. All the workers had been visited by an occupational physician, and then by a psychiatrist, for psychological and psychiatric issues from January 2016 to May 2019. 

### 2.2. Assessment (Definition of Variables)

Data of the sample were retrospectively collected from July to September 2021 by means of the web platform Infoclin, a software used for the management of health clinical data at Sant’Orsola-Malpighi Polyclinic. Only visits for psychic fitness were considered. 

Sociodemographic information about sex, age, type of job, and department where the individuals worked, were collected. Data about the number of visits with the occupational physician, year of the first visit, diagnoses of mental disorder according to the DSM-5 [45], psychotherapeutic and/or pharmacological treatment, fit notes, and limitations at work according to the occupational physician, were reported as well. All subjects received a fit note according to their health status, as follows: fit for work, unfit for work, and fit with limitations. Limitations were prescribed according to orthopedic and/or psychiatric diseases. Regarding limitations at work for orthopedic problems, the occupational medicine Department at Sant’Orsola-Malpighi Polyclinic relies on a categorization from type A to type D based on the “Dictionary of Occupational Title” [46], according to the level of physical demand that workers can tolerate. Indeed, type A means that workers are able to cope with very heavy work: occasionally workers have to move objects > 45 kg, frequently 23 kg, constantly 9 kg; type B means that workers are able to cope with medium work: occasionally workers have to move objects 9–23 kg, frequently 4.5–11.5 kg, constantly 4.5 kg; type C means that workers are able to cope with light work: occasionally workers have to move objects < 9 kg, frequently < 4.5 kg, constantly negligible; type D means that workers are able to cope with very light work: occasionally workers have to move objects < 4.5 kg, frequently negligible, constantly negligible. Limitations related to psychiatric disorders include direct care exemption, night shifts exemption, high-risk area exemption, unfit for a specific department, and exemption from emergency departments.

Data of the sample were gathered by fulfilling a data collection sheet, specifically prepared for the present investigation. Diagnoses, treatments, and limitations were established by a psychiatrist together with an occupational physician. Data were analyzed anonymously according to the Italian data protection regulation (Legislative Decree No. 196/2003). The study was conducted in accordance with the ethical principles of the Declaration of Helsinki [47].

### 2.3. Data Analysis

Since this study follows a case series design, the sample size was not preliminarily calculated [48]. Descriptive and explorative data analyses were performed. Continuous data were expressed as means (±standard deviation, SD) and categorical data were shown as frequencies and percentages. A general linear model (ANOVA) was run to ascertain statistical differences among participants with different DSM-5 psychiatric diagnoses (fixed factor), according to age (dependent variable). Thereafter, Fisher’s exact test applied to contingency tables was used to evaluate the presence of statistical associations between psychiatric diagnoses and kind of job (i.e., physician, other health professionals, healthcare assistant/technician/other), department (i.e., medical departments, administrative departments), and fit notes (i.e., fit without limitations, fit with limitations/other, unfit). Finally, a descriptive analysis was made in order to outline frequencies of participants with the same psychiatric diagnosis/same orthopedic limitation, according to fit notes and psychiatric work limitations.

All calculations were performed using IBM SPSS 26.0 and a *p*-value < 0.05 was considered to be significant.

## 3. Results

### 3.1. Healthcare Workers’ Requests, Sociodemographic Characteristics, Psychiatric Diagnoses, Assigned Doctor’s Fit Notes and Limitations

The sample included 149 healthcare workers (mean age = 48 ± 9.6 years; F = 73.8%), mainly at the department of oncology/hematology (N = 23; 15.4%) (Table 1). The most represented job category was health professionals, who included nurses (N = 79; 53%), physiotherapist (N = 1; 0.7%), dietician (N = 1; 0.7%) and midwife (N = 1; 0.7%), followed by healthcare assistants (N = 35; 23.5%), such as formal carers (Table 1). The total number of psychiatric consults from 2016 to 2019 was 337, with a mean of 2.3 visits (±1.9) per person, and the most frequent psychiatric diagnosis according to DSM-5 was depression (N = 66; 44.3%) (Table 1). According to the doctor’s fit note, 52 (34.9%) workers were judged fit for work without any limitation, whereas 20 (13.4%) received work limitations for orthopedic issues, 42 (28.2%) for psychiatric conditions, and 23 (15.4%) for both orthopedic and psychiatric problems (Table 1). Finally, 9 (6%) workers were judged unfit for the job (Table 1).

### 3.2. Associations between Psychiatric Diagnoses, Sociodemographic Characteristics and Work-Related Variables

ANOVA did not show any significant association between age and psychiatric diagnoses (F = 0.401; *p* > 0.05) (Table 2). Using Fisher’s exact test, significant differences in psychiatric diagnoses according to sex (χ^2^ = 16.739; *p* < 0.01) and fit notes (χ^2^ = 24.181; *p* < 0.01) have been found, whereas no significant association according to a different kind of job and department has been observed. For further details, see Table 2.

### 3.3. Analysis of Frequencies of Participants with Same Psychiatric Diagnosis/Same Orthopedic Limitation, According to Fit Notes and Psychiatric Work-Limitations

Frequencies of participants with the same psychiatric diagnosis/same orthopedic limitation, according to doctor’s fit notes, are reported in Table 3. As to depressive disorder, which represented the commonest psychiatric condition, according to the physician’s judgment, depressed healthcare workers with the same orthopedic limitations encountered very heterogeneous scenarios. Specifically, among 39 (26.2%) depressed participants with type A limitation, 16 (10.7%) were considered fit to work without any limitation, whereas 23 (15.4%) were judged fit to work with psychiatric limitations. Three (2%) depressed subjects with type B limitation were fit to work with both psychiatric and orthopedic limitations. Among 14 (9.4%) depressed workers with type C limitation, 8 (5.4%) were considered fit to work with only orthopedic limitations, whereas 6 (4%) with both psychiatric and orthopedic limitations. Among 5 (3.4%) depressed subjects with type D limitation, 2 (1.4%) were judged fit to work with only orthopedic limitations, and 3 (2%) with both psychiatric and orthopedic limitations. Healthcare workers diagnosed with other psychiatric conditions encountered similar heterogeneous scenarios (Table 3).

Frequencies of participants with the same psychiatric diagnosis/same orthopedic limitation, according to psychiatric limitations to own job, are reported in Table 4. As to depressive disorder, depressed healthcare workers were assigned with a variety of psychiatric limitations, orthopedic issues being equal. Specifically, among 39 (26.2%) depressed subjects with type A limitation, 16 (10.7%) did not have psychiatric limitations, 15 (10.1%) received night shift exemption, 1 (0.7%) both night shift and emergency area exemption, 2 (1.4%) night shift and department activity exemptions, 2 (1.4%) direct care exemption, 2 (1.4%) changed department, and 1 (0.7%) was readdressed to work in outpatient placement. Among 14 (9.4%) depressed healthcare workers with type C limitation, 8 (5.4%) did not have psychiatric limitations, 5 (3.4%) received night shift exemption, and 1 (0.7%) changed department. Among 5 (3.4%) depressed subjects with type D limitations, 2 (1.4%) did not have psychiatric limitations, 1 (0.7%) received night shift exemption, and 2 (1.4%) direct care exemption. Healthcare workers diagnosed with other psychiatric conditions encountered similar heterogeneous scenarios (Table 4).

## 4. Discussion

The aims of this study were to describe the psychiatric clinical picture, according to current nosography, of healthcare workers at Sant’Orsola-Malpighi Polyclinic in Bologna, who required one or more visits to the Occupational Medicine Department from 2016 to 2019, and to understand the clinical procedure used to prescribe fit notes and work limitations according to psychiatric diagnoses, orthopedic limitations being equal.

Firstly, descriptive analyses revealed requests from almost three-quarters of females and a prevalence of nurses unevenly distributed across different departments of the hospital. This is consistent with the literature showing that adult women report a higher prevalence of affective disorders and psychoses than men among healthcare workers and in the general population [49,50]. In line with what has been described in the literature [49], the present study revealed a significant association between sex and psychiatric diagnoses. Indeed, sex could be taken into account as a factor that may influence developing or exacerbating disorders such as depression in vulnerable people. In the present study, no significant association between age and presence or type of diagnosis was found.

In the present investigation, the most frequent diagnosis was depressive disorder. This result is in line with the categorization of mental disorders by Glozier [2], which places depressive disorders in the category of the most frequent psychiatric disorders at work. Although there are numerous studies in the literature that have reported a high level of stress and a higher incidence of mental disorders related to nursing work [12,13,14,15], the results of our study did not support this finding. Indeed, contrary to what was expected [12,13,14,15,16,17,18,20], no significant associations between type of diagnosis and type of job were found, as well as between diagnosis and type of department, despite the fact that in some departments workers are characterized by high work-related stress and consequent mental disorders [21,22,23,24,25,26,27,51].

Furthermore, a descriptive analysis of the frequencies highlighted a great heterogeneity in fit notes and work limitations assigned by the psychiatrist in cooperation with the occupational doctor to categories of workers with the same psychiatric diagnosis (orthopedic limitations being equal). In fact, although some health workers have the same diagnosis, their assigned fit notes and job limitations varied a lot, ranging from complete unrestricted fitness to unfit for the job, from a change in working hours to a direct care exemption, which entails a reorganization of the working environment. It is possible to hypothesize that several factors contributed to the formulation of the psychiatrist’s clinical judgment in order to assign different fit notes and work limitations. These factors might include the worker’s medical history, presence of other comorbidities, course of the disorder including the specific stage (i.e., a person may present a psychiatric diagnosis in its acute, residual, or chronic phase), type, severity and sequence of symptoms, other aspects of daily life and other clinical characteristics resulting in diverse clinical, prognostic, and therapeutic differences among individuals [52]. Indeed, it is not surprising that most of the time clinicians rely not only on explicit evidence from research but also on their own experience and that of their colleagues (tacit knowledge or “mind-lines”) [53]. The standard procedure applied in occupational medicine, as well as in psychiatric settings in general, is thus based most of the time on the psychiatrist’s own clinical judgments following internal clinical parameters, which should be considered as clinimetric parameters. Feinstein [54] proposed a clinimetric approach that is intended to translate this internal judgment into clinical parameters, which could be shared among different clinicians. The clinimetric approach is an integrative model to the psychometric one in the evaluation of psychopathology, with the aim of making the assessment according to a clinical (and not only to a statistical) consistency. It advocates for including within a frame of reference the patient him or herself as a whole, and not simply his/her disorder [54]. It follows that it would be important to conduct a clinimetric assessment that could integrate the problem not only from a categorical nosographic point of view but also from a dimensional perspective considering the various factors mentioned above [55]. 

The literature, in line with the present findings, highlights a lack of standardized guidelines for the assessment of psychic fitness for a specific job and for the assignment of limitations and the fact that effective assessment instruments are not used [40]. The clinimetric approach could fill this gap. Indeed, it emphasizes the importance of including both observer- and self-rated instruments in the clinical assessment process that could allow discriminating between groups of patients with the same clinical diagnosis, physical impairment being equal [56].

The present investigation has some limitations that should be discussed. The first is represented by the cross-sectional design and the preliminary nature of the findings, which were obtained retrospectively by consulting electronic medical records (i.e., the Infoclin platform). Secondly, a convenience sampling method was used, thus limiting the generalizability of study findings. Finally, the third limit of this study is monocentricity, since it included workers from only one hospital located in Northern Italy. Future research should consider larger samples from different hospitals located in different regions in Italy, as well as in different European countries.

## 5. Conclusions

The findings of the present study have shown that a categorical approach in identifying the nuances of the severity of mental illness does not match with the complexity of the human being, and does not match, accordingly, with fit notes and job limitation assignments during the assessment procedure of occupational health visits. This procedure does not allow taking into consideration specific clinical factors that might be associated with a more specific fit note and task limitation assignment. In order to standardize the procedure in occupational medicine settings and translate into practice the psychiatrist’s clinical judgment, further studies are needed to test the usefulness of a clinimetric approach, including not only categorical but also dimensional tools, to assess the course of mental illness, stage of the disorder, level of severity of patient’s clinical picture, and comorbidity. These factors may be discriminatory in both prognosis and treatment of patients and may therefore be more specific and reliable in considering different fit notes and job limitations.

## Figures and Tables

**Table 1:** Socio-demographic characteristics of the participants (N = 149).

	N (%)	Mean (±DS)
**Age** (range 23–65 years old)		48.0 (±9.6)
**Sex**		
Male	39 (26.2)	
Female	110 (73.8)	
**Job**		
Physicians	11 (7.4)	
Other health professionals	82 (55)	
Healthcare assistants	35 (23.5)	
Technicians, other	21 (14.1)	
**Department**		
*Medical departments*		
Oncology/hematology	23 (15.4)	
Women and children	18 (12.1)	
Gatroenterology/endocrinology	18 (12.1)	
Nephrology	17 (11.4)	
Cardiology	14 (9.4)	
Radiology	12 (8.1)	
Emergency	9 (6)	
Head/neck	9 (6)	
Outpatient clinic	8 (5.4)	
Infectivology	3 (2)	
Scheduled hospitalizations	3 (2)	
Test laboratory	1 (0.7)	
*Administrative departments*		
Administrative management	12 (8.1)	
Public relations office	2 (1.3)	
**Number of psychiatric evaluations** (range 1–13 visits)		2.3 (±1.9)
**Year of first psychiatric evaluation**		
2016	43 (28.9)	
2017	39 (26.2)	
2018	41 (27.5)	
2019	26 (17.4)	
**Psychiatric diagnoses**		
None	25 (16.8)	
Depressive disorder	66 (44.3)	
Anxious-depressive disorder	16 (10.7)	
Panic disorder with agoraphobia	13 (8.7)	
Anxiety disorder	7 (4.7)	
Alcohol abuse with/without comorbidity	5 (3.3)	
Bipolar disorder	4 (2.7)	
Post-traumatic stress disorder	3 (2)	
Adjustment disorder	3 (2)	
Eating disorder	2 (1.3)	
Personality disorder	3 (2)	
Obsessive-compulsive disorder	1 (0.7)	
**Fit notes and limitations**		
None	52 (34.9)	
Unfit	9 (6)	
Fit with orthopedic limitations	20 (13.4)	
Fit with psychiatric limitations	42 (28.2)	
Fit with both psychiatric and orthopedic limitations	23 (15.4)	
Not available	3 (2)	

**Table 2:** Associations between psychiatric diagnoses and age, sex, job, department, and limitations.

	None (N = 25)	Mood Disorder (N = 70)	Anxiety Disorder (N = 20)	Anxious-Depressive Disorder (N = 16)	Alcohol Abuse (N = 5)	Stress-Related Disorders (N = 6)	Other (N = 7)		
	Mean ± SD	Mean ± SD	Mean ± SD	Mean ± SD	Mean ± SD	Mean ± SD	Mean ± SD	F	*p*
**Age**	47.4 ± 10.8	48.9 ± 9.4	47.1 ± 9.6	48.3 ± 8.7	49.6 ± 13.8	45.3 ± 7.2	44.4 ± 10.9	0.401	0.877
	**N (%)**	**N (%)**	**N (%)**	**N (%)**	**N (%)**	**N (%)**	**N (%)**	χ^2^ *	***p*** *
**Sex**									
Male	10 (25.6)	14 (35.9)	6 (15.4)	0 (0)	2 (5.1)	3 (7.7)	4 (10.3)	16.739	0.006
Female	15 (13.6)	56 (50.9)	14 (12.7)	16 (14.5)	3 (2.7)	3 (2.7)	3 (2.7)		
**Job**									
Physicians	2 (18.2)	4 (36.4)	1 (9.1)	2 (18.2)	1 (9.1)	0 (0)	1 (9.1)	7.720	NS
Other health professionals	12 (14.6)	40 (48.8)	10 (12.2)	10 (12.2)	2 (2.4)	5 (6.1)	3 (3.7)		
Healthcare assistants, technicians, other	11 (19.6)	26 (46.4)	9 (16.1)	4 (7.1)	2 (3.6)	1 (1.8)	3 (5.4)		
**Department**									
Medical Dept.	23 (17)	63 (46.7)	17 (12.6)	16 (11.9)	5 (3.7)	6 (4.4)	5 (3.7)	5.260	NS
Administrative Dept.	2 (14.3)	7 (50)	3 (21.4)	0 (0)	0 (0)	0 (0)	2 (14.3)		
*Women and children* ^a^	3 (16.7)	9 (50)	2 (11.1)	2 (11.1)	2 (11.1)	0 (0)	0 (0)		
*Cardiology* ^a^	2 (14.3)	8 (57.1)	3 (21.4)	0 (0)	1 (7.1)	0 (0)	0 (0)		
*Radiology* ^a^	2 (16.7)	8 (66.7)	0 (0)	1 (8.3)	0 (0)	1 (8.3)	0 (0)		
*Nephrology* ^a^	4 (23.5)	6 (35.3)	3 (17.6)	3 (17.6)	0 (0)	1 (5.9)	0 (0)		
*Oncology/hematology* ^a^	2 (8.7)	13 (56.5)	1 (4.3)	3 (13)	1 (4.3)	1 (4.3)	2 (8.7)		
*Head/Neck* ^a^	3 (33.3)	3 (33.3)	1 (11.1)	1 (11.1)	0 (0)	1 (11.1)	0 (0)		
*Emergency* ^a^	0 (0)	4 (44.4)	3 (33.3)	2 (22.2)	0 (0)	0 (0)	0 (0)		
*Infectivology* ^a^	0 (0)	1 (33.3)	1 (33.3)	0 (0)	0 (0)	0 (0)	1 (33.3)		
*Gastroenterology/Endocrinology* ^a^	4 (22.2)	7 (38.9)	3 (16.7)	2 (11.1)	0 (0)	1 (5.6)	1 (5.6)		
*Scheduled hospitalizations* ^a^	1 (33.3)	0 (0)	0 (0)	1 (33.3)	0 (0)	1 (33.3)	0 (0)		
*Outpatient clinic* ^a^	2 (25)	3 (37.5)	0 (0)	1 (12.5)	1 (12.5)	0 (0)	1 (12.5)		
*Test laboratory* ^a^	0 (0)	1 (100)	0 (0)	0 (0)	0 (0)	0 (0)	0 (0.0)		
*Administrative management* ^b^	2 (16.7)	5 (41.7)	3 (25)	0 (0)	0 (0)	0 (0)	2 (16.7)		
*Public relations office* ^b^	0 (0)	2 (100)	0 (0)	0 (0)	0 (0)	0 (0)	0 (0)		
**Fit notes**									
Fit without limitations	13 (25)	18 (34.6)	6 (11.5)	5 (9.6)	3 (5.8)	1 (1.9)	6 (11.5)	24.181	0.006
Fit with limitations/other	10 (11.5)	48 (55.2)	12 (13.8)	11 (12.6)	1 (1.1)	5 (5.7)	0 (0)		
Unfit	2 (20)	4 (40)	2 (20)	0 (0)	1 (20)	0 (0)	1 (10)		
*Type B (+other)* ^c^	0 (0)	1 (25)	1 (25)	1 (25)	0 (0)	1 (25)	0 (0)		
*Type C (+other)* ^c^	3 (12.5)	13 (54.2)	3 (12.5)	2 (8.3)	0 (0)	3 (12.5)	0 (0)		
*Type D (+other)* ^c^	1 (33.3)	1 (33.3)	0 (0)	1 (33.3)	0 (0)	0 (0)	0 (0)		
*No night shift (+other)* ^c^	5 (18.5)	17 (63)	3 (11.1)	2 (7.4)	0 (0)	0 (0)	0 (0)		
*No direct care (+other)* ^c^	1 (6.7)	8 (53.3)	1 (6.7)	4 (26.7)	1 (6.7)	0 (0)	0 (0)		
*Change department* ^c^	0 (0)	1 (0.7)	0 (0)	1 (0.7)	0 (0)	1 (0.7)	0 (0)		
*Other* ^c^	0 (0)	1 (33.3)	2 (66.7)	0 (0)	0 (0)	0 (0)	0 (0)		
*Unspecified/sent to commission* ^c^	0 (0)	6 (75)	2 (25)	0 (0)	0 (0)	0 (0)	0 (0)		

* Fisher’s exact test; ^a^ frequencies of each medical department; ^b^ frequencies of each administrative department; ^c^ frequencies of each limitation assigned by the physician.

**Table 3:** Frequencies of participants with the same psychiatric diagnosis/same orthopedic limitation, according to fit notes.

	Fit	Fit with Psychiatric Limitations	Fit with Only Orthopedic Limitations	Unfit
	N (%)	N (%)	N (%)	N (%)
Depressive disorder + Type A	16 (10.7)	23 (15.4)	-	4 (2.7)
Depressive disorder + Type B	-	3 (2.0)	0 (0.0)	
Depressive disorder + Type C	-	6 (4.0)	8 (5.4)	
Depressive disorder + Type D	-	3 (2.0)	2 (1.3)	
Anxious-depressive disorder + Type A	3 (2.0)	3 (2.0)	-	1 (0.7)
Anxious-depressive disorder + Type B	-	2 (1.3)	0 (0.0)	
Anxious-depressive disorder + Type C	-	2 (1.3)	1 (0.7)	
Anxious-depressive disorder + Type D	-	1 (0.7)	1 (0.7)	
Alcohol abuse + Type A	1 (0.7)	1 (0.7)	-	0 (0.0)
Panic disorder with agoraphobia + Type A	8 (5.4)	2 (1.3)	-	1 (0.7)
Panic disorder with agoraphobia + Type B	-	2 (1.3)	0 (0.0)	
Panic disorder with agoraphobia + Type C	-	1 (0.7)	0 (0.0)	
Anxiety disorder + Type A	1 (0.7)	3 (2.0)	-	0 (0.0)
Anxiety disorder handling only with help	-	1 (0.7)	0 (0.0)	
Anxiety disorder + Type C	-	0 (0.0)	2 (1.3)	
Bipolar disorder + Type A	1 (0.7)	1 (0.7)	-	0 (0.0)
Bipolar disorder + Type B	-	0 (0.0)	1 (0.7)	
Bipolar disorder + Type C	-	0 (0.0)	1 (0.7)	
Work-related stress + Type A	0 (0.0)	1 (0.7)	-	1 (0.7)
Work-related stress + Type C	-	0 (0.0)	1 (0.7)	
Insomnia + Type A	-	1 (0.7)	-	0 (0.0)

**Table 4:** Frequencies of participants with same psychiatric diagnosis/same orthopedic limitation, according to psychiatric limitations to work.

	No Psychiatric Limitations	No Night Shift	No Night Shift + Department Activity	No Night Shift + No Direct Care	No Direct Care	Change Department	Outpatient Placement	No Night Shift + No Emergency Area	No Emergency Area
	N (%)	N (%)	N (%)	N (%)	N (%)	N (%)	N (%)	N (%)	N (%)
Depressive disorder + Type A	16 (10.7)	15 (10.1)	2 (1.3)	0 (0)	2 (1.3)	2 (1.3)	1 (0.7)	1 (0.7)	0 (0)
Depressive disorder + Type B	0 (0)	3 (2)	0 (0)	0 (0)	0 (0)	0 (0)	0 (0)	0 (0)	0 (0)
Depressive disorder + Type C	8 (5.4)	5 (3.4)	0 (0)	0 (0)	0 (0)	1 (0.7)	0 (0)	0 (0)	0 (0)
Depressive disorder + Type D	2 (1.3)	1 (0.7)	0 (0)	0 (0)	2 (1.3)	0 (0)	0 (0)	0 (0)	0 (0)
Anxious-depressive disorder + Type A	3 (2)	1 (0.7)	0 (0)	0 (0)	1 (0.7)	0 (0)	0 (0)	0 (0)	1 (0.7)
Anxious-depressive disorder + Type B	0 (0)	1 (0.7)	0 (0)	0 (0)	1 (0.7)	0 (0)	0 (0)	0 (0)	0 (0)
Anxious-depressive disorder + Type C	1 (0.7)	1 (0.7)	0 (0)	0 (0)	0 (0)	0 (0)	0 (0)	1 (0.7)	0 (0)
Anxious-depressive disorder + Type D	1 (0.7)	0 (0)	0 (0)	0 (0)	1 (0.7)	0 (0)	0 (0)	0 (0)	0 (0)
Alcohol abuse + Type A	1 (0.7)	0 (0)	0 (0)	0 (0)	1 (0.7)	0 (0)	0 (0)	0 (0)	0 (0)
Panic disorder with agoraphobia + Type A	8 (5.4)	0 (0)	0 (0)	1 (0.7)	1 (0.7)	0 (0)	0 (0)	0 (0)	0 (0)
Panic disorder with agoraphobia + Type B	0 (0)	0 (0)	0 (0)	0 (0)	0 (0)	1 (0.7)	0 (0)	0 (0)	0 (0)
Panic disorder with agoraphobia + Type C	0 (0)	1 (0.7)	0 (0)	0 (0)	0 (0)	0 (0)	0 (0)	0 (0)	0 (0)
Anxiety disorder + Type A	1 (0.7)	1 (0.7)	0 (0)	0 (0)	1 (0.7)	0 (0)	1 (0.7)	0 (0)	0 (0)
Anxiety disorder handling only with help	0 (0)	1 (0.7)	0 (0)	0 (0)	0 (0)	0 (0)	0 (0)	0 (0)	0 (0)
Anxiety disorder + Type C	2 (1.3)	0 (0)	0 (0)	0 (0)	0 (0)	0 (0)	0 (0)	0 (0)	0 (0)
Bipolar disorder + Type A	1 (0.7)	0 (0)	0 (0)	0 (0)	1 (0.7)	0 (0)	0 (0)	0 (0)	0 (0)
Bipolar disorder + Type B	1 (0.7)	0 (0)	0 (0)	0 (0)	0 (0)	0 (0)	0 (0)	0 (0)	0 (0)
Bipolar disorder + Type C	1 (0.7)	0 (0)	0 (0)	0 (0)	0 (0)	0 (0)	0 (0)	0 (0)	0 (0)
Work-related stress + Type A	0 (0)	1 (0.7)	0 (0)	0 (0)	0 (0)	0 (0)	0 (0)	0 (0)	0 (0)
Work-related stress + Type C	1 (0.7)	0 (0)	0 (0)	0 (0)	0 (0)	0 (0)	0 (0)	0 (0)	0 (0)
Insomnia + Type A	1 (0.7)	1 (0.7)	0 (0)	0 (0)	0 (0)	0 (0)	0 (0)	0 (0)	0 (0)

## Data Availability

The original data analyzed are available from the corresponding author on reasonable request.
